# Role of Solid Additives in Morphological and Structural Optimization of Bulk Heterojunction Organic Solar Cells

**DOI:** 10.3390/ma19071387

**Published:** 2026-03-31

**Authors:** Muhammad Raheel Khan, Bożena Jarząbek, Abid Ullah

**Affiliations:** 1 Centre of Polymer and Carbon Materials, Polish Academy of Sciences, Sklodowskai-Curie 34 Str., 41-819 Zabrze, Poland; 2 Joint Doctoral School, Silesian University of Technology, Akademicka 2A, 44-100 Gliwice, Poland; 3 Mechanical Engineering Department, University of Alberta, Edmonton, AB T6G 2R3, Canada; aullah4@ualberta.ca

**Keywords:** organic solar cells, bulk heterojunction, solid additives, morphology control, phase separation, molecular packing

## Abstract

Additive engineering has become a critical strategy for optimizing the morphology and performance of bulk heterojunction (BHJ) organic solar cells (OSCs), while volatile solid additives have been widely employed to control nanoscale phase separation during film formation. Concerns regarding reproducibility, residual solvent effects, and long-term stability have stimulated increasing interest in non-volatile solid additives. In recent years, solid additive engineering has emerged as a promising approach for modulating molecular packing, regulating phase separation, enhancing charge transport, and improving device stability. However, a systematic analysis of its material design principles and performance impact remains limited. This review summarizes recent progress in solid additive engineering for OSCs, categorizing reported additives into non-volatile, volatile and nanomaterials. The effects of these additives on key photovoltaic parameters, including open-circuit voltage (Voc), short-circuit current density (Jsc), fill factor (FF), and power conversion efficiency (PCE), are comparatively analyzed based on the reported data. Particular emphasis is placed on morphology and structural performance relationships and stability enhancement mechanisms. Finally, current challenges, including the lack of universal molecular design rules and limited mechanistic understanding of additive host interactions, are discussed, and future research directions are proposed. This review aims to provide a comprehensive perspective on the material-level role of solid additives and to guide the rational design of next-generation high-performance and stable organic solar cells.

## 1. Introduction

Organic solar cells (OSCs) have emerged as a promising photovoltaic technology owing to their lightweight nature, mechanical flexibility, semi-transparency, and compatibility with low-temperature solution processing [1,2,3,4,5,6]. Over the past decade, power conversion efficiencies (PCEs) of bulk heterojunction (BHJ) organic solar cells have increased dramatically, surpassing 19% for single-junction devices through molecular engineering and morphology optimization strategies [7]. This rapid progress has been largely driven by the development of non-fullerene acceptors (NFAs), refined donor–acceptor energy-level alignment, and advanced active layer processing techniques.

The bulk heterojunction architecture relies on an interpenetrating donor–acceptor network to facilitate efficient exciton dissociation and charge transport. However, achieving the ideal morphology requires a crystal domain size of (10–20 nm) and a bi-continuous interpenetrating network [8,9]. The morphology of the active layer critically governs exciton diffusion, charge separation efficiency, carrier mobility, and recombination dynamics. Therefore, precise morphology control has become one of the most important research directions in organic photovoltaics. Additive engineering has proven to be a powerful strategy to tailor the active layer nanostructure. Traditionally, high-boiling-point volatile solvent additives such as 1,8-diiodooctane (DIO) have been extensively employed to optimize phase separation and crystallinity in polymer:fullerene and polymer:NFA blends [10,11], and volatile additives are a promising way to improve the performance of organic solar cells [12,13,14]. However, residual solvent traces may negatively impact on device performance [15]. The roles and properties of volatile and non-volatile solid additives are shown in Table 1.

Recently, solid additives have emerged as an alternative and complementary approach for morphology regulation in BHJ solar cells. Unlike volatile solid additives, non-volatile solid additives remain within the active layer during film formation and can modulate molecular packing, crystallization kinetics, phase separation and energetic disorder in a more controlled manner. These materials include pyridine derivatives and organic small molecular materials containing hydroxyl groups [16,17]. Recent studies have demonstrated that solid additives can simultaneously enhance PCE and device stability, addressing a key bottleneck in the commercial translation of OSC technology [18]. In non-fullerene systems, where molecular packing and aggregation behavior are particularly sensitive to processing conditions, solid additives have shown strong capability in fine-tuning domain purity and charge transport pathways [19].

Despite the increasing number of reports, a comprehensive and systematic review focused specifically on solid additive engineering for BHJ active layers remains limited. Most existing reviews primarily discuss solvent additive strategies or general morphology control techniques. Therefore, a detailed analysis of solid additive classification, structure–property relationships, and their impact on optical, electronic, and morphological characteristics is timely and necessary.

In our review, particular emphasis is placed on nanoscale morphology and structural characterization, including techniques such as AFM and analyses related to molecular packing and π–π stacking, in order to clarify the mechanistic role of solid additives in optimizing active layer organization. In addition, this review highlights the influence of solid additives on device stability, which is an essential aspect for practical application but was often treated less systematically in previous reports. Therefore, the novelty of this review lies in providing a more targeted, structure-, morphology-, and stability-based perspective on solid additive engineering in OSCs.

## 2. Non-Volatile Solid Additives

In contrast to volatile systems, non-volatile solid additives remain embedded within the active layer after film formation and thermal treatment. Rather than acting as temporary morphology directors, these additives function as permanent microstructural modulators that can influence molecular packing, crystallinity, the dielectric environment, and even charge transport pathways. Their presence can enhance intermolecular interactions, suppress excessive phase separation, and improve the mechanical robustness of the bulk heterojunction network. Moreover, non-volatile additives may serve additional roles such as trap passivation, dipole formation, and energy-level modulation, thereby contributing simultaneously to morphological and electronic optimization. However, because they persist within the film, careful concentration control is essential to avoid unfavorable aggregation, excessive dilution of the photoactive components, and long-term stability issues.

**Figure 1:**
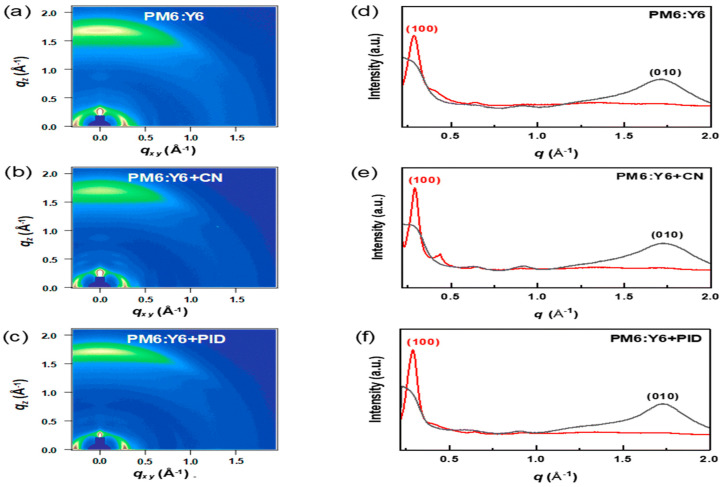
2D GIWAXS patterns of (**a**) PM6:Y6, (**b**) PM6:Y6 + CN, and (**c**) PM6:Y6 + PID; (**d**–**f**) corresponding out-of-plane (black lines) and in-plane (red lines) directions. Reproduced from Ref. [20]. Copyright 2026, Royal Society of Chemistry.

**Figure 2:**
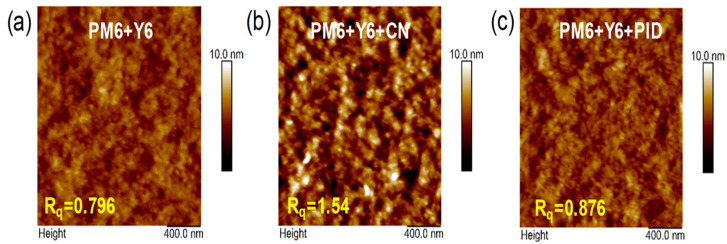
AFM images of (**a**) PM6 + Y6, (**b**) PM6 + Y6 + CN, and (**c**) PM6 + Y6 + PID. Reproduced from Ref. [20]. Copyright 2026, Royal Society of Chemistry.

A notable example of non-volatile solid additive engineering was reported by Choi et al. [20], who designed and synthesized 2-(4-phenoxybenzylidene)-1H-indene-1,3(2H)-dione (PID), which contains DPE and ID moieties. DPE can increase the packing order of the donors while ID enhances the acceptors’ intermolecular π–π stacking. Unlike conventional volatile additives such as 1-chloronaphthalene (CN), PID remains in the active layer after thermal annealing, as confirmed by FT-IR spectroscopy. UV–Vis absorption analysis reveals enhanced vibronic peak intensity of PM6 at 629 nm upon PID incorporation, indicating improved polymer backbone ordering. Simultaneously, the Y6 absorption peak exhibits a 14 nm red shift (to 829 nm), suggesting strengthened intermolecular interactions, whereas CN induces a blue shift, implying disrupted packing. GIWAXS measurements show that all blends maintain a preferential face-on orientation with pronounced (010) π–π stacking along the out-of-plane direction, as shown in Figure 1a–f. Compared with pristine PM6:Y6 (qxy = 0.28 Å^−1^; qz = 1.70 Å^−1^), PID-treated films exhibit increased scattering vectors (qxy = 0.291 Å^−1^; qz = 1.73 Å^−1^), corresponding to reduced lamellar (d100) and π–π stacking (d010) distances, indicating tighter molecular packing. Moreover, the π–π stacking coherence length (CL010) increases significantly, evidencing enhanced crystallinity in the face-on orientation, while CN reduces crystalline coherence. AFM characterization further demonstrates that PID induces controlled nanoscale phase separation with only a slight roughness increase (Rq = 0.87 nm vs. 0.79 nm pristine), whereas CN results in excessive aggregation (Rq = 1.54 nm) which is shown in Figure 2a–c, particularly due to strong Y6 aggregation. As a consequence of the optimized morphology and enhanced molecular ordering, the power conversion efficiency (PCE) improves from 13.5% (pristine) to 15.5% with PID, outperforming CN-processed devices (15%). This work highlights the potential of rationally designed non-volatile solid additives to simultaneously regulate molecular packing, suppress over-aggregation, and stabilize active layer morphology.

**Figure 3:**
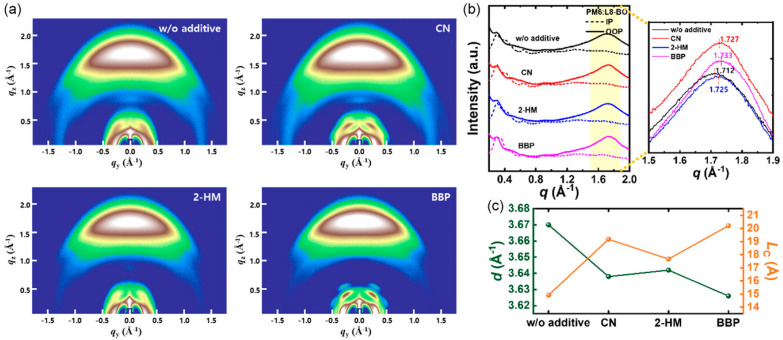
(**a**) GIWAX diffraction pattern, (**b**) line-cut profile in the in-plane (IP) and out-of-plane (OOP) direction, (**c**) comparison of π–π stacking distance, and crystal coherence length (Lc) in the OOP direction for PM6:L8:BO blend films prepared without additive and with CN, 2-HM and BBP respectively [21].

**Figure 4:**
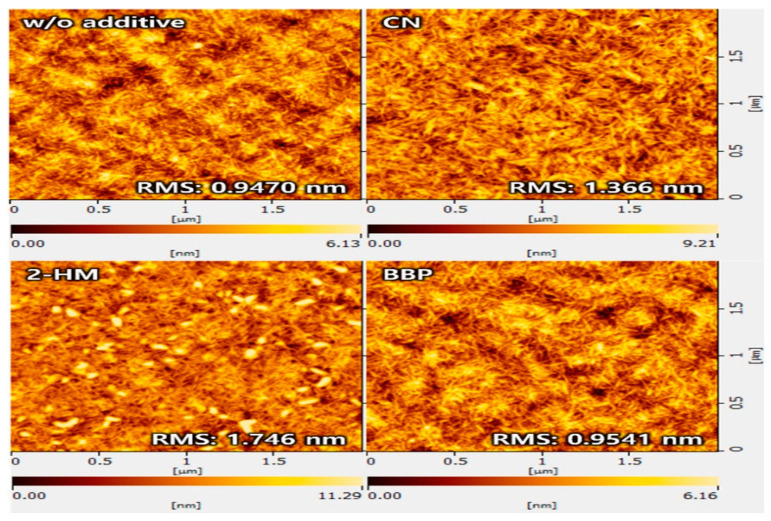
AFM images of the active layer blend films prepared without solid additive and with CN, 2HM and BBP [21].

Kim et al. [21] introduced a non-volatile solid additive to regulate bulk heterojunction (BHJ) morphology in PM6:L8-BO-based organic solar cells. 4-bromobiphenyl (BBP) was compared directly with the solvent additive 1-chloronaphthalene (CN) and the volatile solid additive 2-hydroxy-4-methoxybenzophenone (2-HM) to elucidate the influence of additive volatility on morphology and device performance. Grazing-incidence wide-angle X-ray scattering (GIWAXS) measurements reveal that all blend films with and without additive adopt a predominant face-on molecular orientation as shown in the Figure 3a–c. The introduction of BBP leads to a reduced π–π stacking distance from 3.670 Å (additive-free) to 3.626 Å and an increased out-of-plane crystal coherence length from 14.92 Å to 20.20 Å, indicating enhanced molecular packing and crystallinity compared with both CN- and 2-HM-processed films. Atomic force microscopy (AFM) was employed to examine surface morphology. The PM6:L8-BO blend film without additives exhibits a smooth surface with a root-mean-square (RMS) roughness of approximately 0.95 nm. With CN and 2-HM, the RMS roughness increases to 1.37 nm and 1.75 nm, respectively, indicating the formation of coarser and less uniform morphologies. In contrast, the BBP-treated blend film shows a uniform nanofibrillar texture with an RMS roughness of approximately 0.95 nm, which is shown in Figure 4, indicating suppressed excessive aggregation and improved phase uniformity. The miscibility between PM6 and L8-BO was evaluated using Flory–Huggins interaction parameters. The PM6:L8-BO blend with BBP exhibits a lower interaction parameter (χ ≈ 0.0994 K) than those processed with CN and 2-HM, indicating improved donor–acceptor miscibility. Fourier transform infrared (FTIR) spectroscopy confirms the non-volatile nature of BBP, showing it forms strong intermolecular interactions with both PM6 and L8-BO, which optimize the morphology and crystallinity of the active layer. Thermogravimetric analysis further supports the thermal stability of BBP under device processing conditions. Photovoltaic performance measurements show that the PM6:L8-BO device without additives achieves a power conversion efficiency (PCE) of 15.01%. Devices processed with CN and 2-HM reach PCEs of 16.65% and 16.73%, respectively. In contrast, the device incorporating BBP achieves a significantly higher PCE of 18.11%, with a short-circuit current density of 27.78 mA cm^−2^, an open-circuit voltage of 0.852 V, and a fill factor of 76.52%. The devices treated with BBP solid additives demonstrate the highest thermal stability and retain 80% of their initial PCE after 240 h.

FcF_10_ was introduced into the active layer (PM6:Y6) to regulate intermolecular interactions and optimize nanoscale morphology. The FcF_10_ additive facilitates non-covalent interactions with Y6 such as F···F, F···π and π–π interactions between the Cp and C_6_F_6_ rings in FcF_10_ and the FIC end groups in Y6. Thermogravimetric analysis (TGA) and differential scanning calorimetry (DSC) demonstrate that FcF_10_ exhibits high thermal stability, with the onset of sublimation at approximately 137 °C and negligible mass loss during isothermal holding at 100 °C for 1 h, confirming its non-volatile behavior under device processing conditions. Grazing-incidence wide-angle X-ray scattering (GIWAXS) provides direct evidence of enhanced molecular ordering. In Y6 films, the π–π stacking peak shifts from qz = 1.80 to 1.82 Å^−1^, corresponding to a reduced π–π stacking distance from 3.49 to 3.45 Å, while the coherence length increases from 24.09 to 25.16 Å. For PM6:Y6 blend films, the π–π stacking distance remains at 3.53 Å, but the coherence length increases from 19.13 to 20.94 Å after FcF_10_ addition, indicating larger crystalline domains. Atomic force microscopy (AFM) was used to examine surface morphology. For neat Y6 films, the surface root-mean-square roughness (Rq) increases from 2.46 to 3.85 nm after FcF_10_ incorporation, which is associated with increased crystallinity. In PM6:Y6 blend films, the Rq increases from 2.37 to 3.15 nm with FcF_10_, indicating regulated phase separation and a more favorable nanoscale morphology for charge transport, as reported by the authors. Photovoltaic devices with an inverted architecture (ITO/ZnO/active layer/MoO_3_/Ag) were fabricated. The reference PM6:Y6 device exhibits a power conversion efficiency (PCE) of 15.34%. With 3.75 wt% FcF_10_, the device achieves a higher PCE of 17.00%, with a Voc of 0.85 V, a Jsc of 27.35 mA cm^−2^, and a fill factor of 73.29% [22]. PM6:Y6 devices treated with the FcF_10_ solid additive exhibit enhanced thermal stability, retaining 88% of their initial PCE after prolonged annealing at 85 °C for 360 h.

**Figure 5:**
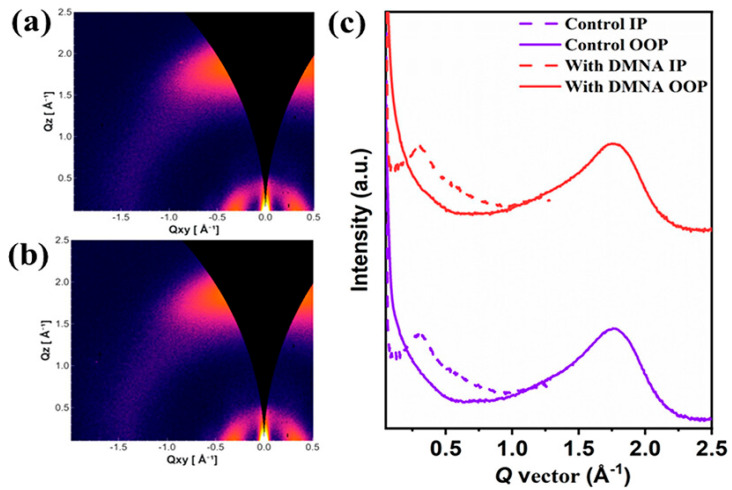
2D GIWAX patterns of (**a**) untreated devices and (**b**) devices with DMNA; (**c**) 1D line-cut profile along the IP and OOP direction. Reproduced with permission from Ref. [23]. Copyright 2026, American Chemical Society.

**Figure 6:**
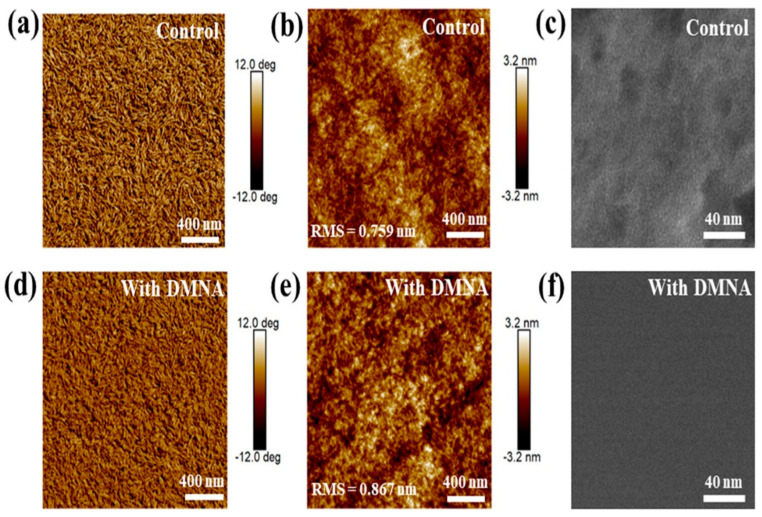
AFM images of: (**a**,**d**) phase images, (**b**,**e**) height images, and (**c**,**f**) TEM images of different blend films treated without and with DMNA. Reproduced with permission from Ref. [23]. Copyright 2026, American Chemical Society.

Liu et al. [23] incorporated 1,4-dimethoxynaphthalene (DMNA), a halogen-free, non-volatile solid additive, to regulate the bulk heterojunction morphology of D18-Cl:N3-based organic solar cells. N3 and DMNA molecules can form a strong intermolecular interaction due to their opposite charge distribution. Thermogravimetric analysis shows that DMNA exhibits a 5% weight loss temperature of approximately 142.2 °C, while differential scanning calorimetry revealed a melting point of 87 °C, confirming that DMNA cannot be removed under typical thermal annealing conditions (80 °C) and therefore remains in the active layer after processing. Grazing-incidence wide-angle X-ray scattering (GIWAXS) measurements reveal that both control and DMNA-treated blend films adopt a dominant face-on molecular orientation which is shown in Figure 5a–c. In the in-plane direction, the (100) diffraction peak remains at Q ≈ 0.30 Å^−1^, while the crystalline coherence length (CCL) slightly increases from 60.585 to 61.717 Å after DMNA addition. In the out-of-plane direction, strong π–π stacking (010) diffraction peaks are observed at Q ≈ 1.75 Å^−1^, with the CCL increasing from 27.085 to 28.411 Å, indicating enhanced π–π stacking coherence and molecular packing induced by DMNA. Atomic force microscopy (AFM) and transmission electron microscopy (TEM), which are shown in Figure 6a–f, were employed to characterize the surface and bulk morphologies of blend films. The additive-free D18-Cl:N3 film exhibits a uniform surface with a root-mean-square (RMS) roughness of 0.759 nm. Upon incorporation of 15 wt % DMNA, the RMS roughness increases to 0.867 nm, indicating stronger molecular assembly and enhanced crystallinity. AFM phase images reveal a well-defined fibrillar network, while TEM images show more pronounced acceptor-rich crystalline regions, suggesting optimized nanoscale phase separation. Photovoltaic measurements demonstrate that the optimized DMNA-treated device achieves a power conversion efficiency (PCE) of 18.61%, compared to 17.21% for the additive-free control device. This enhancement is primarily attributed to simultaneous increases in short-circuit current density (27.74 mA cm^−2^) and fill factor (76.84%), while the open-circuit voltage remains nearly unchanged. The DMNA-treated devices retain 93% of their initial PCE after 250 h while the device without DMNA maintains 88% of its initial PCE.

Three eco-friendly, halogen-free solid additives—4,4′-dihydroxybiphenyl (DBP), 4,4′-dimethylbiphenyl (DMBP), and 2,2′-dihydroxy-4-methoxybenzophenone (DM)—were introduced into the active layer processed from the non-halogenated solvent to enhance the sustainability of organic photovoltaics [24]. Fourier-transform infrared spectroscopy confirms the non-volatile nature of DBP. Characteristic DBP peaks remain visible in PM6:L8-BO blend films even after thermal annealing at 130 °C for 10 min, whereas the characteristic peaks of DMBP and DM disappear after annealing, indicating that DMBP and DM volatilize under these conditions. Grazing-incidence wide-angle X-ray scattering (GIWAXS) measurements show that all PM6:L8-BO blend films adopt a predominant face-on orientation, characterized by strong (010) π–π stacking peaks in the out-of-plane direction. The π–π stacking distance decreases from 3.607 Å (without additives) to 3.564 Å (DBP), 3.572 Å (DMBP), and 3.576 Å (DM). Correspondingly, the coherence length increases from 25.24 Å to 27.72 Å (DBP), 27.32 Å (DMBP), and 26.93 Å (DM), demonstrating the enhanced crystallinity, particularly for DBP-treated films. Atomic force microscopy (AFM) measurements reveal that pristine PM6 films show little morphological change with additives, except for DBP, which yields a more uniform surface. In contrast, L8-BO films exhibit strong additive-dependent morphology variations. L8-BO films with DM show pronounced aggregation, whereas films with DBP display smoother surfaces, indicating that additive volatility strongly influences acceptor morphology. For PM6:L8-BO blend films, the additive-free film exhibits a rough surface with a root-mean-square (RMS) roughness of 2.474 nm, indicating poor miscibility. The incorporation of DBP, DMBP, and DM results in smoother morphologies with RMS values of 1.340 nm, 1.515 nm, and 2.064 nm, respectively. DBP-treated films show the most uniform fibrillar morphology and the smallest fibril width (~19.82 nm). Photovoltaic devices with a conventional architecture of ITO/Br-2EPSe/PM6:L8-BO/PDINN/Ag are fabricated. The device without additives exhibits a power conversion efficiency (PCE) of 15.01%, with a Jsc of 24.00 mA cm^−2^, Voc of 0.832 V, and FF of 75.16%. With DBP, the PCE increases to 17.78%, accompanied by an increased Jsc of 27.18 mA cm^−2^, Voc of 0.845 V, and FF of 77.43%. Devices with DMBP and DM achieve PCEs of 17.13% and 16.46%, respectively [24]. The DBP-treated devices maintain 82% of their initial PCE after 1000 h of storage.

Chloroprene rubber (CR) was introduced as a non-volatile solid additive and plasticizer in D18:L8BO organic solar cells to simultaneously enhance efficiency and mechanical robustness [25]. AFM analysis shows that moderate CR loading maintains uniform fibrillar morphology (Rq ≈ 1.43 nm for control), whereas excessive CR (60 wt%) induces phase separation (Rq ≈ 13.0 nm), correlating with performance deterioration. GIWAXS measurements reveal a predominant face-on orientation with a stable π–π stacking distance of ~3.61 Å, while lamellar coherence length increases with CR addition, indicating enhanced donor ordering. XPS and DFT analyses confirm strong halogen and π–π interactions between CR and D18, validating its non-volatile structural role. Photovoltaic devices with 5 wt% CR achieve an improved PCE of 19.25% (vs. 18.49% control) with an enhanced fill factor and balanced charge mobility, while higher CR loading gradually reduces PCE due to morphological disruption. In ultra-flexible devices, 5 wt% CR yields 16.91% efficiency and significantly improves mechanical stability under cyclic deformation. Overall, CR optimizes morphology, crystallinity and charge transport enabling high efficiency without sacrificing flexibility. The devices treated with 5 wt % CR retain 79.8% of their initial PCE after 5000 cycles.

A novel organic small molecule, BBT-Cl, was employed as a non-volatile solid additive in combination with CN solvent additive to optimize PM6:Y6 organic solar cells [26]. AFM analysis shows that the RMS roughness slightly increases from 1.01 nm (CN) to 1.16 nm (CN + BBT-Cl), accompanied by a larger fibril width, indicating regulated phase separation and improved charge transport. GIWAXS measurements reveal enhanced π–π stacking intensity at ~1.72 Å^−1^ with reduced FWHM (0.187 vs. 0.200), suggesting increased crystallinity without altering molecular orientation. Theoretical calculations were performed by DFT in order to study the intermolecular interactions of PM6:BBT-Cl and Y6:BBT-Cl. The binding energy between PM6 and BBT-Cl is larger than Y6:BBT-Cl and the distance of C···H···F is much smaller than C···H···O. These results suggest that BBT-Cl shows stronger interaction with the acceptor Y6. This result is consistent with the result obtained from GIWAX. Thus, BBT-Cl increases the crystallinity and molecular packing of Y6 and PM6:Y6 blends through intermolecular hydrogen bonds. The strengthened intermolecular interactions, particularly between BBT-Cl and Y6, promote more ordered molecular packing. Consequently, the dual-additive device achieves an improved PCE of 17.73% (Voc = 0.851 V; Jsc = 26.95 mA cm^−2^; FF = 77.3%) compared to 16.48% for CN-only devices. The enhanced Jsc and FF are attributed to higher charge dissociation probability (92.1%) and suppressed bimolecular recombination. Furthermore, reduced residual CN improves operational stability, significantly prolonging device lifetime. Overall, BBT-Cl acts as an effective morphology regulator and crystallinity enhancer, enabling simultaneous efficiency and stability improvement.

Shao et al. [27] developed double-hydrogen-bond (2H-bond) non-volatile solid additives (2H-TR, 2H-TC6R, 2H-TCi8R, and 2H-TOC8R) for PTB7-Th/PC60BM solar cells processed with 3.0 wt% DIO. FTIR confirms strong hydrogen-bond interaction between the amide groups of the additives and the fluorine atoms of PTB7-Th, indicating that the additives remain in the film and stabilize morphology. AFM shows smooth surfaces with RMS values of 1.38–1.49 nm after additive incorporation, while the control film exhibits severe aggregation after heating (RMS increases to 5.43 nm). With 2H-TOC8R, the morphology remains stable after annealing at 100 °C, preserving a uniform bi-continuous network. The additives improve the charge balance significantly, with μh/μe increasing from 0.54 (control) to 0.99 for 2H-TOC8R, confirming enhanced transport. As a result, PCE increases from 6.02% (control) to 7.18% (Voc = 0.80 V; Jsc = 12.94 mA cm^−2^; FF = 69.55%), and thermal stability improves markedly due to suppressed PC60BM aggregation.

An integrated omnidirectional design strategy was employed to develop a non-volatile solid additive, PyMC5, for high-efficiency PM6:Y6 and PM6:L8-BO organic solar cells [28]. Thermogravimetric analysis confirms its non-volatile nature with high decomposition temperatures, ensuring retention during device fabrication. AFM analysis shows that the pristine PM6:Y6 film exhibits an RMS roughness of 1.12 nm, while PyDC5- and PyMC5-treated films display reduced RMS values of 0.889 nm and 0.975 nm, respectively, indicating an optimized and more uniform morphology. GIWAXS measurements reveal dominant face-on orientation with π–π stacking distances of 3.626 Å (control), 3.756 Å (PyDC5), and 3.608 Å (PyMC5), demonstrating more compact molecular packing for PyMC5-treated films. The improved crystallinity and favorable miscibility enhance charge transport and suppress recombination. Consequently, PM6:L8-BO devices incorporating PyMC5 achieve a PCE of 19.52% (Voc = 0.904 V; Jsc = 27.25 mA cm^−2^; FF = 79.10%), surpassing the control devices (18.13%). Reduced non-radiative voltage loss (ΔE_3_ = 0.227 eV) and improved charge-transfer lifetimes further contribute to performance enhancement. Additionally, PyMC5-treated devices exhibit improved photo- and thermal stability, maintaining over 90% of initial efficiency under stress conditions.

Two coumarin-derived solid additives, volatile C5 and non-volatile C6, were developed to systematically compare their working mechanisms in organic solar cells [29]. UV–Vis absorption and TGA analyses confirm that C5 volatilizes upon thermal annealing, whereas C6 remains in the active layer, verifying its non-volatile nature. AFM measurements show that the pristine PM6:Y6 blend exhibits an RMS roughness of 1.15 nm, while C6-treated films display a slightly increased but more favorable fibrillar morphology with an RMS of 1.24 nm; in contrast, C5-treated films exhibit over-aggregation. GIWAXS results reveal a dominant face-on orientation with a (010) π–π stacking peak at 1.740 Å^−1^ for the control film; upon C6 addition, the π–π stacking distance becomes 3.57 Å with an enhanced coherence length of 31.29 Å, indicating improved molecular crystallinity and packing. Although C5 further tightens the π–π stacking to 3.55 Å with a coherence length of 32.48 Å, excessive aggregation limits morphological optimization. Consequently, D18:L8-BO devices treated with C6 achieve a superior PCE of 20.32% (Voc = 0.932 V; Jsc = 26.79 mA cm^−2^; FF = 81.40%), outperforming C5-treated (19.55%) and additive-free (18.85%) devices as shown in Table 2 [29]. PM6:Y6 devices treated with C5 and C6 retain 84% and 87% of their initial PCE after TA of 230 h at 80 °C.

### Nanomaterials as Solid Additives

Nanomaterial-based additives represent an advanced strategy in bulk heterojunction (BHJ) organic solar cells, where nanoscale inorganic or carbon-based materials are incorporated to simultaneously tailor morphology and electronic properties. Due to their high surface area, tunable surface functionality, and unique electrical characteristics, nanomaterials can serve as crystallization promoters, charge transport enhancers, and trap passivation centers within the active layer. Unlike molecular additives, nanomaterials such as metal oxide nanoparticles, quantum dots, carbon nanotubes, and graphene derivatives can introduce additional percolation pathways for charge carriers while influencing donor–acceptor phase separation at the nanoscale. Their incorporation may enhance exciton dissociation efficiency, improve carrier mobility, and suppress recombination losses. However, achieving uniform dispersion and preventing aggregation remain critical challenges. Surface modification or functionalization is often required to ensure compatibility with the polymer–acceptor matrix, thereby enabling controlled morphology evolution without compromising optical absorption or film homogeneity.

Nor et al. [30] report the incorporation of cadmium sulfide (CdS) quantum dots (QDs) into the photoactive layer of a quaternary organic solar cell with the architecture FTO/ZnO nanorods/P3HT:PC61BM:CQDs:CdS/Ag. FESEM analysis shows a slight increase in active layer thickness without severe film disruption at optimal 4 wt% loading. Photovoltaic measurements show that the optimized 4 wt% CdS device achieves a power conversion efficiency (PCE) of 2.87%, representing a 12% improvement compared to the control device (2.58%). The improvement is primarily driven by an increase in short-circuit current density from 11.80 to 12.60 mA/cm^2^, along with slight enhancements in open-circuit voltage (0.46 to 0.48 V) and fill factor (0.47 to 0.48). Additionally, series resistance decreases (14.8 to 12.5 Ω·cm^2^) and shunt resistance increases (165 to 254 Ω·cm^2^), indicating improved charge extraction and reduced recombination. IPCE measurements further confirm enhanced photocurrent generation, with peak IPCE increasing from approximately 67% to 71%.

Phosphorus-doped carbon quantum dots (P-CQDs) were incorporated as a third-component additive into the P3HT:PCBM bulk heterojunction active layer to enhance morphology, structural ordering, and photovoltaic performance [31]. Morphological analysis using atomic force microscopy (AFM), revealed that low P-CQD concentrations (1–3 vol%) significantly reduced surface roughness from 15.49 nm (pristine) to 8.90 nm (1%) and 6.28 nm (3%), indicating smoother and more homogeneous films. Grain size distribution increased concurrently, suggesting improved phase separation and enhanced charge transport pathways. However, higher concentrations (5–7 vol%) led to agglomeration, increased roughness (up to 31.65 nm), and disrupted interpenetrating networks, negatively affecting carrier transport. Structural characterization via X-ray diffraction (XRD) demonstrated enhanced polymer chain ordering upon P-CQD incorporation. The characteristic P3HT (100) peak (~5.5°) and PCBM peak (~21.5°) showed increased intensity compared to the pristine film, confirming improved crystallinity. Crystallite size increased from 8.7 nm (without additive) to approximately 14–16 nm at optimal concentrations, with the highest value observed at 3 vol% P-CQDs (~16.2 nm). This enhanced crystallinity supports more efficient charge separation and transport. Photovoltaic measurements confirmed that morphology and structural improvements translated into device performance enhancement. The optimized device containing 1 vol% P-CQDs achieved a power conversion efficiency (PCE) of 2.482%, compared to 2.189% for the reference device. This improvement was primarily driven by increased short-circuit current density (Jsc increased from 6.80 to 7.46 mA cm^−2^) and improved fill factor (from 53.83% to 55.34%), while Voc showed slight enhancement. The optimized device also exhibited reduced series resistance (17.10 Ω cm^−2^) relative to the pristine device (22.31 Ω cm^−2^), indicating improved charge extraction. Overall, the study demonstrates that controlled incorporation of P-CQDs enhances crystallinity, phase separation, and charge transport, whereas excessive loading deteriorates morphology and photovoltaic performance, highlighting the delicate balance between nanostructuring and electronic optimization in ternary organic solar cells.

**Figure 7:**
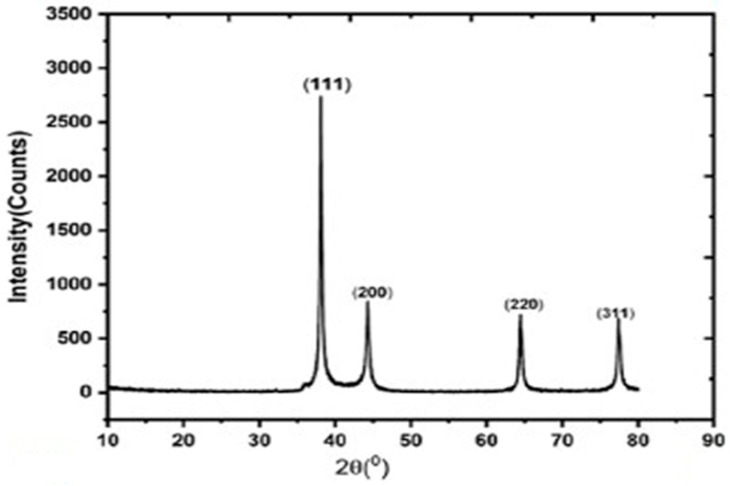
XRD spectra of Ag-NRs [32].

**Figure 8:**
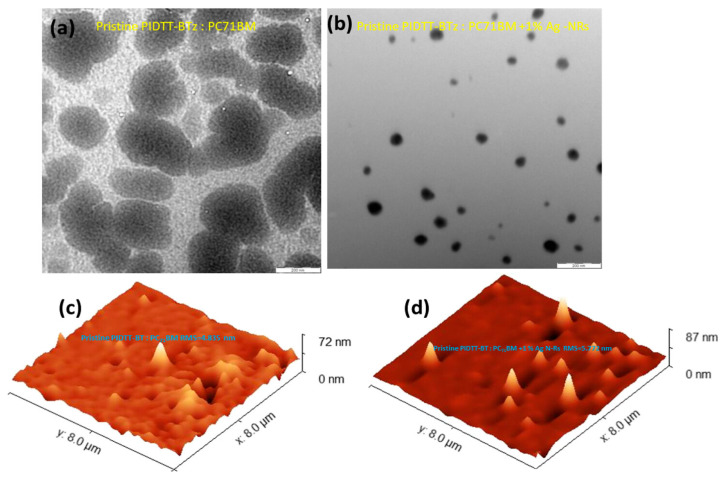
TEM image of the active layer (**a**) without Ag-NRs and (**b**) with 1% Ag-NRs, and AFM images of the active layer (**c**) without Ag-NRs and (**d**) with 1% Ag-NRs [32].

Waketola et al. [32] introduced silver nanorods (Ag-NRs) into a polymer-based active layer, PIDTT-BTz:PC71BM, to enhance light harvesting via localized surface plasmon resonance (LSPR). X-ray diffraction (XRD) analysis confirmed a face-centered cubic (fcc) crystalline structure of Ag with characteristic diffraction peaks at 38.12°, 44.32°, 64.48°, and 77.40°, corresponding to (111), (200), (220), and (311) planes, which are shown in Figure 7, and an average crystallite size of approximately 21 nm. The TEM image of plasmonic devices with 1% Ag-NRs, which is shown in Figure 8b, reveals that NPs are well dispersed and do not form aggregates which lower charge generation. Atomic force microscopy (AFM) revealed only a slight increase in surface roughness (from 4.83 nm to 5.78 nm), which is shown in Figure 8c,d, indicating minimal disruption of film morphology. Photophysical studies showed stronger photoluminescence quenching upon Ag-NR incorporation, indicating enhanced exciton dissociation. The maximum carrier generation rate (Gmax) increased from 8.35 × 10^21^ to 9.06 × 10^21^ cm^−3^ s^−1^, and exciton dissociation probability improved from 89.48% to 93.65%. Space-charge-limited current (SCLC) measurements revealed nearly doubled charge mobility and reduced series resistance. Consequently, short-circuit current density increased from 11.92 to 13.58 mA cm^−2^, fill factor improved from 39.50% to 42.65%, and power conversion efficiency rose from 3.94% to 4.93%, representing a 26% enhancement. Overall, optimized Ag-NR incorporation enhanced light harvesting and carrier dynamics without significantly disturbing morphology, while excessive loading induced aggregation and recombination losses.

Akhatova et al. [33] investigate the incorporation of MoS_2_ nanoparticles into the P3HT:PC61BM bulk heterojunction (BHJ) active layer of inverted organic solar cells (FTO/ZnO/P3HT:PC61BM:MoS_2_/PEDOT:PSS/Ag) to enhance morphology, crystallinity, and photovoltaic performance. Atomic force microscopy (AFM) phase images revealed that pristine P3HT films exhibit a lamellar morphology, while MoS_2_ incorporation increases lamellar length and phase contrast, indicating improved polymer chain ordering and crystallinity. In P3HT:PC61BM blends, MoS_2_ nanoparticles were mainly embedded within the film bulk without severely disrupting morphology at the optimized concentration. X-ray diffraction (XRD) analysis showed enhancement of the P3HT (100) diffraction peak at 2θ ≈ 5.24°, confirming improved crystallinity, while a new diffraction peak at 2θ ≈ 14.36° verified successful incorporation of MoS_2_. Photovoltaic measurements revealed significant improvement at optimal 0.5 wt% MoS_2_ loading: short-circuit current density increased from 5.01 to 9.1 mA cm^−2^, open-circuit voltage from 0.46 to 0.76 V, fill factor from 0.39 to 0.43, and power conversion efficiency from 0.90% to 2.97%, representing a threefold enhancement.

Synergistic incorporation of isopropanol (IPA) and carbon quantum dots (CQDs) significantly improved morphology, structural ordering and one-sun photovoltaic performance of inverted P3HT:PCBM solar cells [34]. UV–Vis absorption spectra revealed a slight increase in optical absorption upon IPA incorporation, which further increased when CQDs were introduced. Cross-sectional FESEM analysis showed that the active layer thickness increased from 191 ± 10 nm (pristine) to 198 ± 10 nm (IPA) and 216 ± 9 nm (CQDs/IPA), contributing to enhanced light harvesting. AFM analysis demonstrated a substantial increase in surface roughness from 4.85 nm (pristine) to 13.6 nm (IPA) and 17.4 nm (CQDs/IPA). The increased roughness enhanced internal reflection and scattering, promoting higher optical absorption. Electrical analysis showed that IPA reduced series resistance (Rs) and slightly increased shunt resistance (Rsh), while CQDs further decreased Rs (to 27.35 Ω·cm^2^) and significantly increased Rsh (to 667.13 Ω·cm^2^), reflecting improved charge extraction and suppressed recombination. Energy-level alignment indicates that CQDs provide favorable electron transport pathways due to suitable conduction band alignment with PCBM and ZnO. Photovoltaic performance under one-sun illumination improved markedly: PCE increased from 0.50% (pristine) to 0.76% (IPA) and further to 1.00% (2 wt% CQDs/40 vol.% IPA), representing nearly 100% improvement relative to pristine. Jsc increased from 2.67 mA cm^−2^ to 3.96 mA cm^−2^, FF improved from 39.6% to 50.1%, and Voc increased slightly from 0.48 V to 0.51 V. Overall, the study demonstrates that IPA primarily improves phase separation and P3HT crystallinity, while CQDs enhance exciton dissociation and charge transport, leading to significant performance enhancement under standard illumination.

Fluorinated MWCNTs were incorporated into PCDTBT:PC60BM organic solar cells to enhance morphology and stability [35]. Raman and SEM confirmed successful fluorination and nanotube integrity. F-MWCNTs modified the active layer and reduced the energetic disorder for charge transfer. Photovoltaic performance improved by ~20% at optimal 1 wt% loading (PCE from 3.11% to 3.50%) due to increased Jsc and FF, while mobility remained unchanged. Light-induced EPR showed reduced energetic disorder (37 meV to 12.5 meV), indicating improved nanomorphology. Long-term stability over 1200 h was significantly enhanced, demonstrating that F-MWCNTs reinforce and stabilize the bulk heterojunction structure.

Plasmonic gold nanoparticles were introduced into ITO-free inverted organic solar cells based on AZO/ZnO/PTB7:PC71BM/MoO3/Ag architecture, to enhance efficiency and stability [36]. Structural characterization of the AZO cathode revealed a hexagonal wurtzite crystal structure with a dominant (103) diffraction peak at 62.85°. The AZO film exhibited a sheet resistance of ~20 Ω/sq and ~80% visible transmittance, with a sharp UV cutoff near 380 nm due to its 3.26 eV bandgap, confirming its UV filtering capability. UV–Vis absorption of Au nanoflowers displayed dual plasmonic peaks at 588 nm (core resonance) and 748 nm (petal resonance), enabling broadband localized surface plasmon resonance (LSPR) enhancement. Photovoltaic characterization showed that the fabricated AZO-based inverted OSC achieved a PCE of 6.19%, slightly lower than the ITO control device (6.83%). However, upon incorporation of Au nanoflowers into the active layer, PCE increased to 7.01%, corresponding to a ~13% enhancement relative to the AZO device. The improvement was primarily driven by increased Jsc (15.20 to 16.15 mA cm^−2^) and enhanced fill factor (67.89% to 71.18%), while Voc remained nearly constant (~0.61 V). EQE measurements demonstrated an enhanced spectral response across 400–780 nm due to plasmon-induced near-field enhancement. Overall, the study demonstrates that replacing ITO with AZO improves device stability, while plasmonic Au nanoflowers enhance light absorption, exciton generation, and carrier collection without disturbing device energetics.

**Figure 9:**
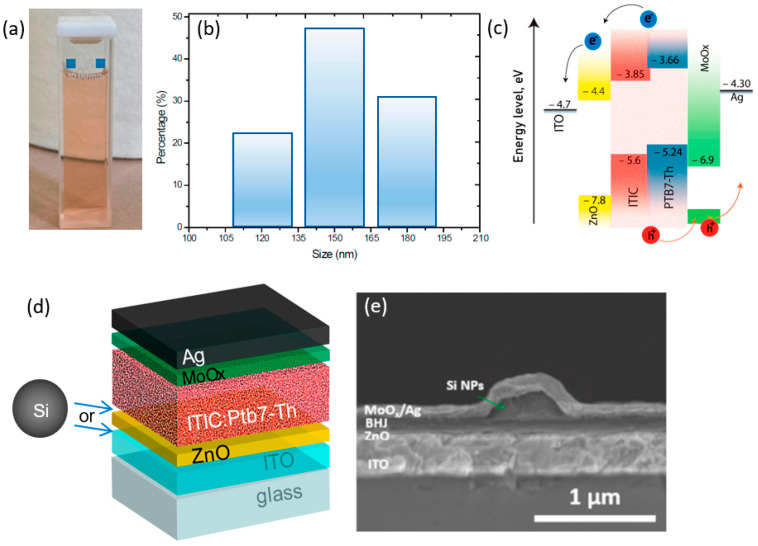
(**a**) Photo of a colloidal NP solution. (**b**) Size distribution of the nanoparticles in the solution. (**c**) Energy band diagram of the device. (**d**) Architecture of the solar cell. (**e**) SEM image of the obtained device covered with NPs [37].

Sandzhieva et al. [37] investigated the incorporation of optically resonant silicon nanoparticles (Si NPs) in solution form, which are shown in Figure 9a, and the size of the NPs was 150 nm, which is shown in Figure 9b. The NPs were incorporated into inverted PTB7-Th:ITIC bulk heterojunction organic solar cells, as shown in Figure 9c,d, to enhance light harvesting via Mie resonances. Cross-sectional SEM analysis confirmed localized nanoparticle incorporation between transport layers and the active layer without disrupting the multilayer device architecture (ITO/ZnO/PTB7-Th:ITIC/MoOx/Ag), as shown in Figure 9e. Uniform dispersion was achieved via spin-coating at low concentrations, while higher concentrations resulted in nanoparticle aggregation and morphological non-uniformity. The optimized configuration (p-type Si NPs, 140–180 nm, and concentration 3 × 10^−4^ mol L^−1^) increased power conversion efficiency from approximately 6.0% (reference) to 7.5%, corresponding to nearly 25% enhancement. This improvement resulted from simultaneous increases in short-circuit current density, open-circuit voltage, and especially fill factor. Higher nanoparticle concentration (6 × 10^−4^ mol L^−1^) caused aggregation, reduced morphological uniformity, and caused performance decline. The enhancement mechanism was attributed primarily to Mie-resonant light trapping and near-field optical amplification, with p-type doping additionally supporting favorable charge transport. Overall, the study demonstrates that dielectric silicon nanoparticles provide a low-loss, plasmon-free light-management strategy for enhancing organic solar cell efficiency when size, concentration, and placement are carefully optimized.

The morphological properties of the active layer play a decisive role in determining the photovoltaic performance of the studied organic solar cells. TEM analysis reveals that pristine POxT:PC71BM film exhibits large acceptor-rich domains with poor bicontinuity, limiting efficient charge transport pathways, while brominated POxT-Br further disrupts polymer packing, resulting in unfavorable phase separation and reduced fill factor [38]. In contrast, thiol-functionalized POxT-SH forms a well-defined bi-continuous interpenetrating network with narrower fibrillar domains, facilitating exciton diffusion and balanced charge extraction. This morphological improvement is reflected in the AFM roughness values, where POxT-SH shows a very smooth surface (0.57 nm) compared to POxT (2.10 nm) and POxT-Br (1.54 nm), indicating more controlled nanoscale phase separation. Upon incorporation of 0.171 wt% Au nanoparticles, the roughness increases to 2.94 nm, suggesting enhanced nanostructuring and increased interfacial area for light interaction. The gold nanoparticles, stabilized through Au–S interactions with the thiol groups, promote more uniform polymer alignment and improved percolation pathways while simultaneously inducing plasmonic light enhancement. As a result, the short-circuit current density increases significantly from 3.49 mA/cm^2^ (POxT) to 6.52 mA/cm^2^ (POxT-SH with Au NPs), accompanied by an improvement in fill factor to 64.5%. Therefore, the efficiency enhancement is not solely due to plasmonic effects but primarily arises from nanoparticle-assisted morphological optimization that improves charge generation, transport, and extraction within the bulk heterojunction.

The incorporation of Au and Ag nanoparticles significantly modified the structural and morphological properties of the P3HT:PCBM active layer, which directly influenced photovoltaic performance. In [39] the XRD analysis shows that the pristine P3HT:PCBM blend exhibited a characteristic diffraction peak at 2θ ≈ 5.37°, corresponding to its semicrystalline ordering, while additional peaks at 38.4° and 44.3° confirmed the incorporation of face-centered cubic Au and Ag nanoparticles within the active layer. The presence of these metallic reflections suggests modified molecular packing and partial enhancement of structural ordering. AFM measurements further demonstrated that nanoparticle incorporation increased surface roughness due to nanoparticle-induced surface protrusions and localized aggregation, with Ag (0.5 wt%) films showing slightly higher RMS roughness compared to Au (1.5 wt%) films. Moderate roughness was found to enhance light scattering and promote localized surface plasmon resonance (LSPR), thereby increasing optical absorption and exciton generation. At optimized concentrations, the balance between improved crystallinity and controlled nanoscale morphology facilitated enhanced charge transport and reduced recombination losses. This structural optimization resulted in improved fill factor and open-circuit voltage, leading to an increase in power conversion efficiency from 2.11% in the undoped device to 3.11% (Au, 1.5 wt%) and 3.20% (Ag, 0.5 wt%) as shown in Table 3. Nevertheless, excessive nanoparticle loading induced aggregation, which disrupted morphology and adversely affected device performance. Overall, the study demonstrates that controlled plasmonic nanoparticle incorporation simultaneously modifies crystallinity, surface morphology, and optical field distribution, collectively contributing to enhanced photovoltaic characteristics.

## 3. Volatile Solid Additives

In bulk heterojunction (BHJ) organic solar cells, precise control over nanoscale morphology is essential to achieve efficient exciton dissociation and balanced charge transport. While solvent engineering has long been used to tailor phase separation, the incorporation of solid additives has emerged as an effective strategy to further optimize molecular packing, crystallinity, and domain purity. Among these strategies, volatile solid additives offer a unique advantage: they temporarily participate in morphology regulation during film formation and are subsequently removed through thermal annealing or post-treatment. This transient interaction enables enhanced molecular ordering and improved donor–acceptor interpenetrating networks without leaving residual impurities that could negatively affect long-term device stability.

**Figure 10:**
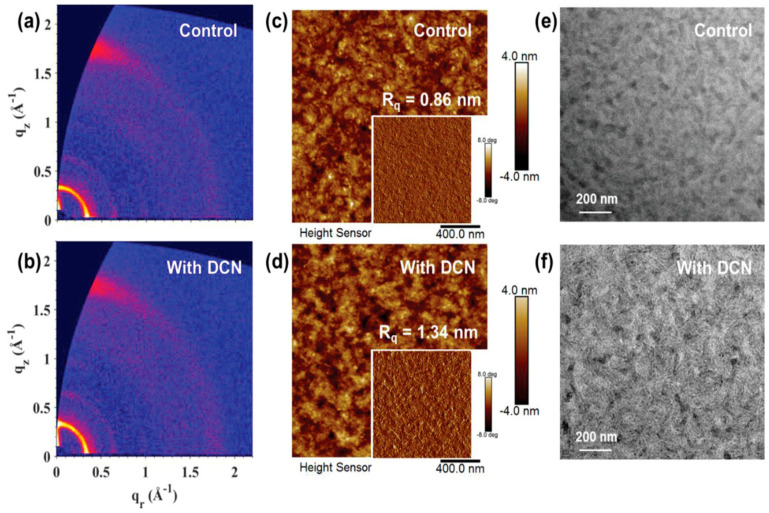
(**a**) 2D GIWAX image of control blend (**b**) 2D GIWAX image of the DCN blend (**c**) AFM image of control blend (**d**) AFM image of the DCN blend (**e**) TEM image of control blend (**f**) TEM image of the DCN blend. Reproduced from Ref. [40]. Copyright 2026, Wiley-VCH.

A volatile solid additive, 1,8-dichloronaphthalene (DCN), was introduced into Tz6T:eC9-4F all-small-molecule organic solar cells to enhance efficiency and stability [40]. TGA shows that DCN starts evaporating above 100 °C, and FTIR confirms that its characteristic peaks disappear after annealing at 160 °C, proving its volatile nature and complete removal from the active layer. GIWAXS measurements show enhanced crystallinity with an increased lamellar coherence length from 13.5 nm to 14.6 nm and slightly improved π–π stacking as shown in Figure 10a,b. The improved molecular ordering promotes better charge transport and reduces bimolecular recombination. AFM images reveal that the control film has an Rq of 0.86 nm, while the DCN-treated film shows a higher Rq of 1.34 nm, as shown in Figure 10c,d, indicating stronger aggregation and clearer phase separation. As evidenced by TEM Figure 10e,f, the blend processed with DCN exhibits well-defined fiber-like nanostructures, demonstrating enhanced donor-acceptor phase separation. As a result, the PCE increases from 14.9% (Voc = 0.860 V; Jsc = 25.75 mA cm^−2^; FF = 67.2%) to 16.0% (Voc = 0.863 V; Jsc = 26.12 mA cm^−2^; FF = 71.0%). The DCN-treated devices also retain over 90% of their initial PCE after 3000 h storage and 500 h thermal or light stress, confirming improved morphological stability.

**Figure 11:**
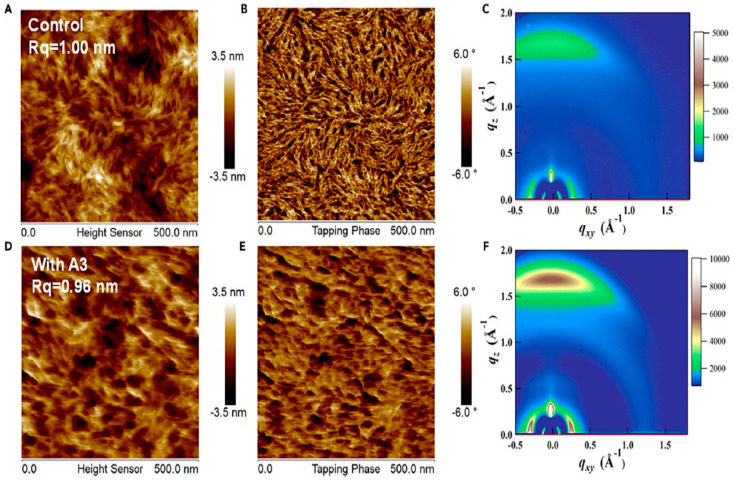
(**A**) AFM height image of PM6:Y6 blend film without A3 additive, (**B**) AFM phase image of PM6:Y6 blend film without A3 additive, (**C**) 2D GIWAX image of PM6:Y6 without A3 additive, (**D**) AFM height image of PM6:Y6 with A3 additive, (**E**) AFM phase image of PM6:Y6 with A3 additive, and (**F**) 2D GIWAX image of PM6:Y6 with A3 additive [42].

A novel volatile solid additive, 2-hydroxy-4-methoxybenzophenone (2-HM), was introduced into PM6:Y6 series solar cells (PM6:Y6, PM6:BTP-eC9, and PM6:L8-BO) to regulate morphology [41]. TGA shows significant weight loss above 80 °C and FTIR confirms the disappearance of its characteristic peaks after thermal annealing, proving complete volatilization with no residue. AFM reveals that the pristine Y6 film exhibits an RMS of ~1.56 nm, while 2-HM treatment induces rougher yet more ordered crystalline grains; in blends, RMS slightly increases but domain distribution becomes more favorable after annealing. GIWAXS shows predominant face-on orientation with π–π stacking around q ≈ 1.7 Å^−1^, and after annealing, tighter π–π stacking and enhanced coherence length are observed. GISAXS analysis indicates reduced correlation length (~25.7 nm) and optimized domain size (~14.7 nm), confirming improved phase purity. The additive strengthens molecular ordering, especially for Y6, leading to enhanced carrier mobility and suppressed trap-assisted recombination. Consequently, PCE improves from 15.31% (PM6:Y6 control) to 17.01%, and up to 18.85% for PM6:L8-BO with 2-HM, mainly due to a higher Jsc and FF. The morphology evolution clearly links tighter stacking and optimized phase separation to improved charge transport and reduced recombination. A σ-hole-containing volatile solid additive, 1,4-diiodotetrafluorobenzene (A3), was incorporated into PM6:Y6 organic solar cells [42]. TGA and FTIR confirm that A3 fully volatilizes after thermal annealing at 110 °C, proving its volatile nature. AFM shows similar surface roughness for control (Rq = 1.00 nm) and A3-treated films (Rq = 0.96 nm), as shown in Figure 11A,B,D,E, but the treated film exhibits a more interconnected nanoscale network, which is beneficial for charge transport. GIWAXS reveals dominant face-on orientation in both films, with the π–π stacking peak shifting from qz = 1.69 Å^−1^ (3.717 Å) to 1.70 Å^−1^ (3.696 Å), as shown in Figure 11C,F. The coherence length increases from 26.75 Å to 27.63 Å. These results indicate enhanced molecular packing and crystallinity without excessive aggregation. The improved ordering promotes balanced charge transport (μh/μe reduces from 2.33 to 1.22) and suppresses recombination. Consequently, PCE increases from 14.8% (Voc = 0.86 V; FF = 68.4%) to 16.5% (Voc = 0.82 V; FF = 76.1%; Jsc = 26.5 mA cm^−2^). The A3-treated devices maintain 97.5% of their initial efficiency after 360 h storage, which is higher than their control counterparts, with 95.1% as their average PCE.

**Figure 12:**
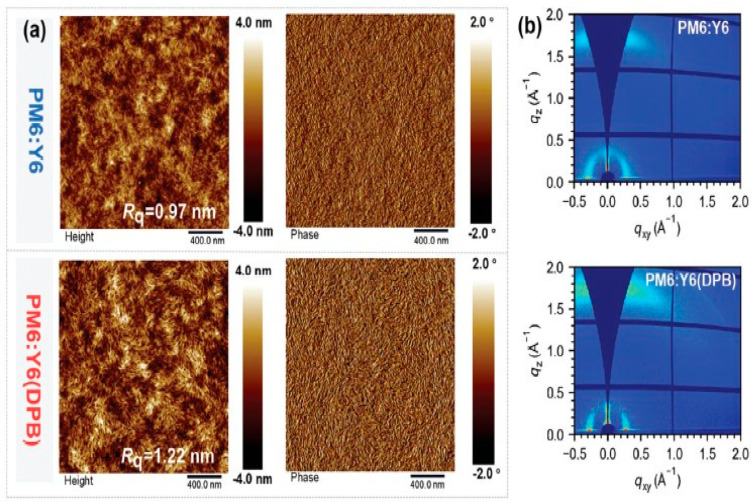
(**a**) AFM and phase image of PM6:Y6 with and without DPB treatment; (**b**) 2D GIWAX image of PM6:Y6 with and without DPB treatment. Reproduced from Ref. [43]. Copyright 2026, Wiley-VCH.

A non-halogenated and twisted volatile solid additive, 1,4-diphenoxybenzene (DPB), was introduced into PM6:Y6 organic solar cells to optimize morphology [43]. TGA and in situ FTIR confirm that DPB volatilizes above 80 °C and its characteristic peak at 1492 cm^−1^ disappears after annealing, proving complete removal from the active layer. AFM shows the control PM6:Y6 blend film has an Rq of 0.97 nm, while DPB-treated film increases to 1.22 nm as shown in Figure 12a, indicating enhanced crystallinity and optimized phase separation. GIWAXS reveals dominant face-on orientation with the π–π stacking distance decreasing from 3.64 Å (q = 1.73 Å^−1^) to 3.57 Å (q = 1.76 Å^−1^), as shown in Figure 12b, and coherence length increasing from 15.83 Å to 18.37 Å, confirming tighter molecular packing. The improved packing enhances charge transport (μe/μh becomes more balanced from 1.55 to 1.12) and suppresses recombination. As a result, PCE improves from 15.54% (Voc = 0.886 V; Jsc = 25.32 mA cm^−2^; FF = 69.8%) to 17.45% (Voc = 0.836 V; Jsc = 27.42 mA cm^−2^; FF = 76.1%).

A volatile solid additive, 1-bromo-2-methoxynaphthalene (B-2MN), was introduced into PM6:L8-BO sequentially deposited organic solar cells. TGA shows a 5% weight loss at 165 °C, and FTIR confirms that the characteristic peak of B-2MN disappears after thermal annealing, verifying its complete volatilization [44]. GIWAXS measurements show a predominant face-on orientation, with the π–π stacking peak shifting from 1.762 to 1.786 Å^−1^ (d-spacing reduces from 3.566 to 3.518 Å) and coherence length increasing from 2.333 to 2.525 nm, confirming enhanced crystallinity. The tighter stacking and optimized vertical phase separation improve and balance charge transport (μh/μe ≈ 1.06) while suppressing trap-assisted recombination. Transient studies reveal faster exciton dissociation and a higher contribution from CT-mediated pathways. Consequently, the binary device achieves a PCE of 19.53% (Voc = 0.881 V; Jsc = 27.14 mA cm^−2^; FF = 81.68%), and the ternary device reaches 20.50%. Overall, B-2MN improves molecular ordering, optimizes phase separation, enhances charge transport, and delivers high efficiency with superior thermal stability. The B-2Min-treated devices retain 92% of their initial PCE after 1000 h. Walia et al. [45] incorporated a volatile solid additive, 1,4-diiodobenzene (DIB), into a PM6:Y6-based active layer with Ga-doped ZnO as the electron transport layer. FTIR confirms that DIB completely evaporates from the active layer without thermal annealing, as its characteristic peaks disappear, proving its volatile nature. AFM analysis shows that the DIB-treated film exhibits an RMS of 2.213 nm compared to 1.915 nm for the CN-treated control, indicating stronger aggregation and improved vertical phase alignment. The morphology suggests enhanced crystallization and better donor–acceptor interpenetrating networks without oversized domains. The optimized morphology enhances charge transport, increases the built-in potential from 0.67 V to 0.85 V, and suppresses recombination losses. As a result, PCE improves from 14.94% for the control device to 16.67% for the DIB-treated device (Voc = 0.82 V; Jsc = 28.88 mA cm^−2^; FF = 71.92%). The performance enhancement is directly linked to improved stacking, balanced mobility, and efficient charge extraction after additive volatilization. The DIB-treated devices retain 92% of their initial PCE after 30 days of storage.

**Figure 13:**
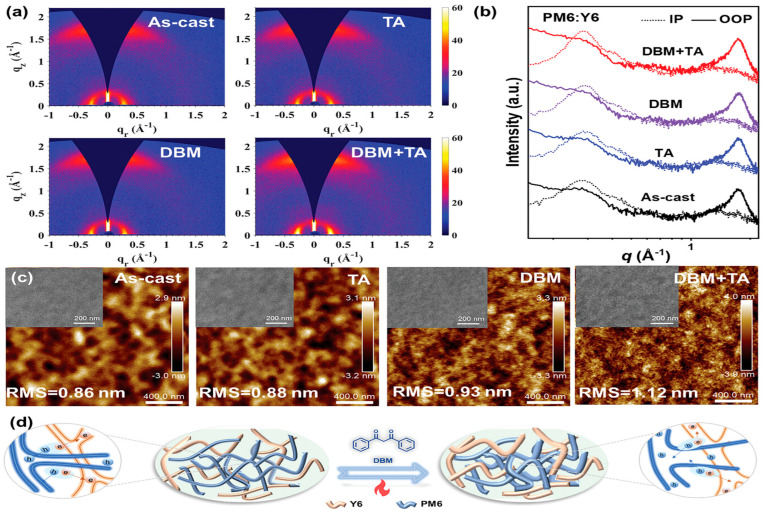
(**a**) GIWAXS patterns. (**b**) Line-cut profiles for PM6:Y6 blend films prepared under various processing conditions. (**c**) AFM and TEM images of various PM6:Y6 blend films. (**d**) Schematic diagrams of PM6:Y6 films with and without DBM + TA treatments. Reproduced from Ref. [46]. Copyright 2026, Wiley-VCH.

A halogen-free volatile solid additive, dibenzoylmethane (DBM), was introduced into PM6:Y6 organic solar cells to regulate molecular stacking and film formation [46]. FTIR spectra confirm its volatility after thermal annealing at 90 °C, indicating complete removal from the film. AFM images show that the as-cast blend exhibits a smooth surface with RMS = 0.86 nm, while DBM and DBM + TA films display clearer fibrous networks with slightly increased RMS values of 0.93 and 1.12 nm as shown in Figure 13c,d, suggesting enhanced molecular ordering and optimized phase separation. GIWAXS results reveal predominant face-on orientation, with a reduced π–π stacking distance from 3.62 Å (as-cast) to 3.60 Å and significantly increased coherence length from 29.4 Å to 36.2 Å after DBM + TA treatment, as shown in Figure 13a,b. The strengthened π–π interaction and higher crystallinity promote charge mobility and suppress recombination. Consequently, the DBM + TA device achieves a record PCE of 18.7% (Voc = 0.830 V; Jsc = 28.7 mA cm^−2^; FF = 78.1%), compared to 15.5% for the as-cast device. The performance improvement directly originates from improved stacking, extended exciton diffusion, balanced transport, and efficient charge extraction after additive volatilization.

Haris et al. [47] incorporated a volatile solid additive, 1,4-Dibromobenzene (DBrB), into the active layer system of PM6:Y6-Bo, and its volatility was confirmed by FTIR and TGA after thermal annealing at 130 °C. AFM analysis showed that the RMS increased from 1.10 nm (control) to 1.35 nm with DBrB, indicating improved crystallinity and clearer phase separation. The treated film formed a well-defined bi-continuous interpenetrating network with optimized domain size. GIWAXS results revealed stronger face-on orientation and tighter π–π stacking (Qz = 1.71 Å^−1^ vs. 1.61 Å^−1^ for control). The π–π coherence length increased from 24.62 Å to 40.60 Å, confirming enhanced molecular ordering. Overall, crystallinity improved without excessive aggregation. The optimized morphology enhanced charge transport, reduced recombination, and balanced mobility. Consequently, the PCE increased from 15.1% (Voc = 0.82 V; Jsc = 24.4 mA cm^−2^; FF = 74.7%) to 17.0% (Voc = 0.84 V; Jsc = 26.2 mA cm^−2^; FF = 76.8%).

Wang et al. [48] designed two volatile solid additives, HBT-1 and HBT-2, for D18:L8-BO:PY-C11 organic solar cells. TGA and FTIR confirm their volatility, as the characteristic peaks disappear completely after annealing at 100 °C for 5 min. AFM shows RMS values of 0.874 nm (control), 0.815 nm (HBT-1), and 0.937 nm (HBT-2), where HBT-2 forms a more pronounced fibrillar morphology with optimized phase separation. GIWAXS reveals predominant face-on orientation in all films, but HBT-2 significantly enhances lamellar and π–π diffraction intensity. The π–π coherence length of D18 increases from 22.27 Å (control) to 31.24 Å with HBT-2, confirming improved crystallinity and tighter stacking. This enhanced ordering promotes balanced mobility (μh/μe ≈ 1.02), faster charge transfer, and suppressed recombination. As a result, PCE improves from 17.83% (Voc = 0.934 V; Jsc = 26.57 mA cm^−2^; FF = 71.83%) to 20.01% (Voc = 0.910 V; Jsc = 27.41 mA cm^−2^; FF = 80.23%), mainly due to the higher Jsc and FF driven by optimized morphology and molecular packing.

Song et al. [49] introduced 2-methoxynaphthalene (2-MN) as a green and volatile solid additive in PM6:PY-DT all-polymer solar cells. Optical microscopy and FT-IR confirm that 2-MN completely volatilizes after thermal annealing at 80 °C, as its characteristic peaks disappear. AFM shows RMS values of 0.79 nm (control), 1.48 nm (CN), and 1.97 nm (2-MN), where 2-MN forms a multi-scale fibrillar network with optimized phase separation. GIWAXS reveals dominant face-on orientation with enhanced (100) lamellar and (010) π–π stacking intensities after 2-MN treatment. The coherence lengths increase to 141.3 Å (PM6) and 58.9 Å (PY-DT), indicating improved crystallinity. This ordered packing enables balanced charge transport (μh/μe ≈ 1.10) and suppressed recombination. As a result, PCE improves from 14.47% (control) and 16.61% (CN) to 17.32% (Voc = 0.951 V; Jsc = 23.84 mA cm^−2^; FF = 76.4%) and 17.03% when processed with o-xylene. The 2-MN-treated devices retain 80% PCE after 380h.

An asymmetric volatile process-aid solid, 1,3-dibromo-5-chlorobenzene (DBCl), was introduced into PM6:Y6 organic solar cells to effectively regulate morphology and molecular packing [50]. TGA and XPS analyses confirm that DBCl is highly volatile and is completely removed after thermal annealing at 75 °C, verifying its true process-aid nature. AFM results show that the roughness of the Y6 film increases from 1.05 nm to 2.31 nm after DBCl treatment, indicating stronger aggregation. In the blend film, the RMS slightly increases from 0.98 nm to 1.35 nm, suggesting optimized phase separation without excessive aggregation. GIWAXS measurements reveal enhanced face-on orientation and tighter molecular packing in the PAS-treated blend, with a π–π stacking distance of 3.71 Å and a coherence length of 22.09 Å, confirming the improved crystallinity of Y6. The improved molecular ordering enhances electron mobility, reduces trap-assisted recombination, and promotes more balanced charge transport. As a result, the PAS-treated device achieves a PCE of 18.5% with Voc ≈ 0.85 V, Jsc ≈ 25.8 mA cm^−2^, and an excellent FF of 78.5% as shown in Table 4 [50]. Overall, the PAS strategy improves crystallinity, optimizes phase separation and vertical distribution, and enables more efficient charge generation, transport, and extraction. The PAS-treated devices retain 80% PCE as compared to the control devices.

## 4. Stability Enhancement by Using Volatile and Non-Volatile Solid Additives

Recent studies have demonstrated that solid additive engineering is an effective strategy for mitigating burn-in degradation and improving the thermal, photo-, storage, and operational stability of organic solar cells. Both volatile and non-volatile solid additives suppress performance decay by limiting molecular diffusion, reducing trap generation, and stabilizing charge extraction under prolonged stress.

Kim et al. [51] reported 9,10-phenanthrenequinone (PQ) as a non-volatile, non-halogenated solid additive for enhancing the stability of PM6:Y6 organic solar cells. Using ISOS-compliant testing, PQ-based unencapsulated devices retained 93% of their initial efficiency after 160 h of thermal aging at 85 °C under a nitrogen environment, exhibiting negligible burn-in degradation. In contrast, devices processed with volatile solvent additives showed pronounced early-stage performance losses. PQ incorporation also improved photostability and shelf stability under both nitrogen and ambient conditions. The enhanced stability was attributed to suppressed molecular reorganization, reduced trap formation during aging, and stabilized charge extraction, highlighting the effectiveness of non-volatile solid additives for long-term device reliability. The incorporation of a perfluorophenyl-functionalized ferrocene derivative (FcF_10_) further improved the thermal stability of PM6:Y6 devices. Devices containing FcF_10_ retained 88% of their initial efficiency after 360 h of aging at 85 °C, which was attributed to suppressed trap-assisted recombination and enhanced thermal robustness during long-term operation [22]. Eco-friendly solid additive strategies processed with non-halogenated solvents have also proven effective for stability improvement. Among the investigated additives, 4,4′-dihydroxybiphenyl (DBP), a non-volatile solid additive, enabled superior stability, with devices retaining approximately 82% of their initial efficiency after 1000 h of dark storage under nitrogen and over 73% after 200 h under both thermal (70 °C) and photostability testing. In contrast, devices processed with the conventional volatile additive CN exhibited rapid degradation within 48 h [24]. Beyond thermal and storage stability, operational durability under realistic working conditions has been significantly improved using non-volatile solid additives. For example, Y6 employed as a solid additive in all-polymer PM6:PY-DT devices delivered exceptional operational stability, achieving a T80 lifetime of 1180 h under continuous one-sun illumination with maximum power point tracking [52].

Song et al. [53] reported a series of multifunctional non-volatile solid additives for enhancing the stability of PM6:PY-C11 all-polymer solar cells. Among them, DTC-C12 enabled outstanding photostability, with devices achieving a T80 lifetime of 1120 h under continuous one-sun illumination using maximum power point tracking, compared to approximately 500 h for additive-free devices. The DTC-C12-based devices also exhibited suppressed efficiency decay under prolonged illumination and improved mechanical durability, retaining 93.6% of their initial efficiency after 3000 bending cycles.

Low-volatility, fused-ring solid additives, including 3,6TTBr and 1,5-dibromonaphthalene, were introduced to enhance the intrinsic stability of PM6:L8-BO-based organic solar cells. While explicit ISOS aging tests were not reported, devices incorporating these additives exhibited a significantly reduced trap density and energetic disorder, along with prolonged exciton lifetimes and suppressed non-radiative recombination. The low-volatility nature of the additives enabled sustained regulation of molecular organization, limiting molecular reorganization and trap growth [54]. Benzoylacetone (BA) was introduced as a highly volatile solid additive to enhance the photostability of PM6:Y6 organic solar cells. Under continuous one-sun illumination at 25 °C in nitrogen, BA-processed devices retained about 80% of their initial efficiency after 220 h and showed substantially slower degradation than devices processed with chloronaphthalene. The improved stability was attributed to the complete removal of BA from the active layer, which suppressed burn-in behavior, reduced trap formation, and stabilized charge extraction, demonstrating the benefit of residue-free volatile solid additives for long-term device stability [55]. Xu et al. [56] reported polycyclic aromatic compounds, including fluorene, dibenzothiophene, and dibenzofuran, as volatile solid additives for enhancing the stability of PM6:L8-BO organic solar cells. Devices incorporating these additives exhibited suppressed burn-in degradation and significantly improved thermal and shelf stability compared to additive-free devices, with dibenzofuran delivering the most stable performance. The improved stability was attributed to transient additive sublimation that increased the glass-transition temperature of the photoactive layer, limited molecular reorganization, and reduced trap-assisted degradation, highlighting the effectiveness of volatile solid additives for residue-free stability enhancement.

Solid additive-assisted stability has also been demonstrated in systems emphasizing long-term storage and operational robustness. For instance, BCN-based D18:L8-BO devices retained over 96% of their initial efficiency after 960 h of storage and exhibited improved stability under MPP operation [57]. Han et al. reported dibenzoylmethane (DBM) as a halogen-free volatile solid additive to enhance the stability of PM6:Y6 organic solar cells. DBM-assisted devices exhibited excellent thermal stability, retaining 80% of their initial efficiency after 200 h at 85 °C, and outstanding storage stability with 95% retention after over 1000 h in nitrogen and an extrapolated T80 approaching 5000 h. Under maximum power point tracking, DBM-processed devices also showed markedly improved photostability with suppressed burn-in degradation. The enhanced stability was attributed to residue-free additive volatilization, reduced trap-assisted recombination, and stabilized charge transport during long-term operation [46]. The incorporation of volatile and non-volatile solid additives has emerged as an effective strategy to enhance the operational stability of PTQ10:Y6 organic solar cells. Under accelerated thermal aging at 150 °C, devices with non-volatile solid additives retained nearly 90% of their initial efficiency after 20 h, compared to only 45% for additive-free devices, while volatile additives delivered intermediate stability. Importantly, solid additives also reduced performance scattering by over 40%, highlighting improved processing reliability. The enhanced stability was attributed to suppressed molecular diffusion, reduced trap formation, and optimized solvent–additive synchronization that minimized void formation and stabilized charge transport during aging [58].

Tarique et al. [45] reported 1,4-diiodobenzene (DIB) as a highly volatile solid additive to enhance the stability of non-fullerene organic solar cells. DIB-treated PM6:Y6 devices retained approximately 92% of their initial efficiency after 30 days of storage in nitrogen and exhibited reduced burn-in degradation compared to devices processed with chloronaphthalene. The improved stability was attributed to complete additive volatilization, which suppressed residual-induced degradation, reduced trap-assisted recombination, and stabilized charge extraction, underscoring the advantage of residue-free volatile solid additives for long-term device stability. Rational molecular design of non-volatile solid additives has recently enabled simultaneous ultra-high efficiency and long-term stability. In this regard, PyMC5-based devices retained over 83% of their initial efficiency after 500 h of continuous illumination and approximately 90% after 500 h of thermal aging at 65 °C [28]. Finally, the record of thermal stability was achieved using non-halogenated, non-volatile solid additives such as PID. Under ISOS-D-2 aging conditions at 85 °C, PID-based PM6:Y6 devices retained 98% of their initial efficiency after 1000 h without burn-in loss, which is shown in Table 5 [20].

## 5. Future Research Directions and Prospectives

Despite the promising advancements achieved through solid additive engineering in bulk heterojunction (BHJ) organic solar cells (OSCs), the field remains in an early developmental stage and requires systematic investigation to transition from empirical optimization to rational material design. One of the most critical challenges is the absence of universal molecular design principles that can predict additive behavior within donor–acceptor blends. Current studies often rely on chemical intuition or structural similarity when selecting additives; however, quantitative correlations between additive molecular properties, such as polarity, dipole moment, π-conjugation length, and hydrogen-bonding capability, and solubility parameters and resulting nanoscale morphology are still insufficiently established. Future research should therefore focus on constructing comprehensive structure–morphology performance relationships that enable predictive additive design. Incorporating theoretical calculations, such as density functional theory and molecular dynamics simulations, may provide deeper insight into intermolecular interactions and miscibility behavior, allowing for more targeted additive synthesis. In addition to rational design, a deeper mechanistic understanding of additive-induced morphological evolution is urgently needed. Most existing investigations rely on ex situ characterization techniques, which provide limited information about the dynamic processes occurring during film formation and device operation. The integration of in situ and operando characterization methods, including real-time grazing-incidence wide-angle X-ray scattering, in situ X-ray diffraction, and time-resolved spectroscopy, would enable direct observation of crystallization kinetics, phase separation dynamics, and molecular packing rearrangements. Such studies could clarify whether solid additives function primarily as crystallization regulators, tie-chain promoters, trap passivators, or phase stabilizers and how these roles evolve over time. A comprehensive mechanistic framework will be essential for optimizing additive concentration, processing conditions, and material compatibility.

Long-term operational stability represents another critical area requiring systematic attention. Although many reports demonstrate improved power conversion efficiency upon additive incorporation, fewer studies address durability under realistic thermal, illumination, and humidity conditions. Solid additives are frequently assumed to enhance morphological robustness; however, additive migration, phase segregation, and potential chemical degradation under prolonged stress remain poorly understood. Future work should therefore incorporate standardized stability protocols and employ depth-profiling and compositional analysis techniques to monitor additive distribution over time. Understanding the relationship between additive chemistry and morphological stability will be crucial for developing materials that simultaneously achieve high efficiency and long device lifetime. Scalability and environmentally responsible processing must also be considered to bridge the gap between laboratory-scale demonstrations and industrial implementation. Most solid additive studies are conducted using spin-coating under controlled laboratory conditions, which may not directly translate to large-area manufacturing techniques such as blade coating, slot-die coating, or roll-to-roll processing. Variations in drying kinetics and film thickness during scalable fabrication can significantly alter phase separation behavior and additive effectiveness. Therefore, future research should systematically evaluate additive performance under industrially relevant deposition methods and explore compatibility with non-halogenated, environmentally benign solvents.

## 6. Conclusions

Solid additive engineering has become an effective strategy for improving the performance of bulk heterojunction organic solar cells. Unlike volatile solid additives, non-volatile solid additives remain in the film after processing. This enables better control over morphology and long-term stability. Solid additives regulate molecular packing, crystallinity, and phase separation in the active layer. They help to optimize nanoscale morphology and improve donor–acceptor interfaces. As a result, the charge transport becomes more efficient and the recombination losses are reduced. These improvements lead to enhanced open-circuit voltage, short-circuit current density, and fill factor. Consequently, the overall power conversion efficiency increases. In addition, solid additives improve morphological stability, which is essential for practical applications. Despite this progress, challenges remain. The interaction between additives and host materials is not yet fully understood. Advanced characterization techniques and in situ studies are needed to clarify the underlying mechanisms. Clear design rules for the selecting solid additives must also be established. In the future, solid additive engineering can be combined with ternary systems, non-fullerene acceptors, and interfacial optimization strategies.

## Figures and Tables

**Table 1:** Summary of the roles and properties of volatile and non-volatile solid additives.

Aspect	Volatile Solid Additive	Non-Volatile Solid Additive
Primary mode of action	Mainly kinetic control of thin-film formation	Mainly thermodynamic/persistent control of final morphology
Volatilization	Easily vaporizes	Does not easily vaporize
Removal	Removal via annealing	Remains in the active layer
Residue	No residue	Leaves residue, can affect stability
Boiling point	Relatively lower	Higher
Main function	Transient morphology control (during film formation)	Long-term morphology control

**Table 2:** Summary of the photovoltaic parameters by using non-volatile solid additives.

Active Layer	Additive	Voc (V)	Jsc (mA/cm^2^)	FF (%)	PCE (%)	Ref
PM6:Y6	w/o	0.80	24.2	69	13.5	[20]
	CN	0.83	24.7	72	15.0	
	PID	0.83	25.5	74	15.5	
PM6:L8-BO	w/o	0.84	25.41	69.81	15.01	[21]
	CN	0.85	26.39	74.13	16.65	
	2-HM	0.81	27.51	74.96	16.73	
	BBP	0.85	27.78	76.52	18.11	
PM6:Y6	w/o	0.84	25.50	71.74	15.34	[22]
	Fc-F10 (3.75 wt%)	0.85	27.35	73.29	17.00	
D18-Cl:N3	w/o	0.87	26.48	74.30	17.21	[23]
	DMNA (15 wt%)	0.87	27.74	76.86	18.61	
PM6:L8-BO	w/o	0.83	24.00	75.16	15.01	[24]
	DBP	0.84	27.18	77.43	17.78	
	DMBP	0.83	26.91	76.74	17.13	
	DM	0.82	26.07	76.38	16.46	
D18:L8BO	w/o	0.90	23.40	68.95	14.65	[25]
	CR	0.91	26.46	79.50	19.25	
PM6:Y6	0.5% CN	0.84	25.80	75.76	16.48	[26]
	0.2% CN + 10% BBT-Cl	0.85	26.95	77.3	17.73	
PTB7-Th:PC60BM	w/o	0.80	12.08	62.29	6.02	[27]
	2H-TR	0.80	12.43	63.34	6.32	
	2H-TC6R	0.80	12.40	65.50	6.49	
	2H-TCi8R	0.81	12.61	65.68	6.71	
	2H-TOC8R	0.80	12.94	69.55	7.18	
PM6:Y6	w/o	0.84	26.30	72.19	15.98	[28]
	PyMC5	0.86	26.82	76.65	17.72	
PM6:L8BO	w/o	0.88	26.62	77.38	18.13	
	PyMC5	0.90	27.25	79.10	19.52	
PM6:Y6	w/o	0.85	25.50	72.82	15.91	[29]
	C5	0.85	26.69	76.82	17.61	
	C6	0.86	27.17	77.15	18.12	
D18-L8BO	w/o	0.92	25.84	78.55	18.85	
	C5	0.93	26.39	79.63	19.55	
	C6	0.93	26.79	81.40	20.32	

**Table 3:** Summary of the photovoltaic parameters of OSCs using nanomaterials as non-volatile solid additives.

Active Layer	Additive	Voc (V)	Jsc (mA/cm^2^)	FF (%)	PCE (%)	Ref
P3HT:PC61BM	w/o	0.46	11.80	47	2.58	[30]
	CdS QDs	0.48	12.60	48	2.87	
P3HT:PCBM	w/o	0.59	6.8	53.83	2.18	[31]
	P-CQDs	0.60	7.46	55.34	2.48	
PIDTT-BTz:PC71BM	w/o	0.83	11.92	39.50	3.94	[32]
	Ag-NRS	0.85	13.58	42.65	4.93	
P3HT:PC61BM	w/o	0.46	5.01	39	0.9	[33]
	MoS_2_	0.76	9.1	43	2.97	
P3HT:PC61BM	w/o	0.48	2.67	39.6	0.50	[34]
	40 vol % IPA	0.47	3.63	44.6	0.76	
	2 wt% CQDS/40 vol % IPA	0.51	3.96	50.1	1.00	
PCDTBT:PC60BM	w/o	0.98	8.61	38.1	3.18	[35]
	F-MWCNTS	0.97	9.00	41.9	3.67	
PTB7:PC71BM	w/o	0.62	16.10	68.42	6.83	[36]
	Au-assisted AZO	0.61	16.15	71.18	7.01	
PTB7-Th:ITIC	w/o	--------	---------	------	6.00	[37]
	Si NPS				7.5	
POxT:PC71BM	w/o	0.70	3.49	52.8	1.29	[38]
POxT-SH:PC71BM	% 0.171 AuNPs	0.78	6.52	64.5	3.29	
P3HT:PCBM	w/o	0.48	17.06	45	2.11	[39]
	1.5 wt% Au	0.56	11.11	62	3.11	
	0.5 wt% Ag	0.52	13.19	50	3.20	

**Table 4:** Summary of the photovoltaic parameters of OSCs using volatile solid additives.

Active Layer	Additive	Voc (V)	Jsc (mA/cm^2^)	FF (%)	PCE (%)	Ref
Tz6T:ec9-4F	w/o	0.86	25.75	67.2	14.9	[40]
	DCN	0.863	26.12	71.0	16.0	
PM6:Y6	w/o	0.85	25.12	71.6	15.31	[41]
	2-HM	0.83	26.82	75.7	17.01	
PM6:Y6	w/o	0.86	25.29	68.40	14.8	[42]
	A3	0.82	26.50	76.05	16.5	
PM6:Y6	w/o	0.88	25.32	69.8	15.74	[43]
	DPB	0.83	27.42	76.1	17.45	
PM6:L8-BO	B-2MN	0.88	27.14	81.68	19.53	[44]
PM6:D18:L8:BO	B-2MN	0.90	27.77	82.04	20.50	
ZnO/PM6:Y6	CN	0.82	26.43	68.44	14.94	[45]
Ga: ZnO/PM6:Y6	CN	0.82	26.62	69.13	15.43	
ZnO/PM6:Y6	DIB	0.82	27.76	69.88	16.08	
Ga: ZnO/PM6:Y6	DIB	0.82	28.88	71.92	16.67	
PM6:Y6	w/o	0.85	25.9	70.0	15.5	[46]
	DBM + TA	0.83	28.7	78.01	18.7	
PM6:Y6-Bo	w/o	0.82	24.4	74.7	15.1	[47]
	DBrB	0.84	26.2	76.8	17.00	
D18:L8-BO:PY-C11	w/o	0.93	26.57	71.83	17.83	[48]
	HBT-1	0.91	26.48	77.42	18.74	
	HBT-2	0.91	27.41	80.23	20.01	
PM6:PY-DT	w/o	0.95	23.17	65.4	14.47	[49]
	CN	0.95	23.54	74.2	16.61	
	2-MN	0.95	23.84	76.4	17.32	
PM6:Y6	w/o	0.86	24.2	69.5	14.5	[50]
	PAS	0.85	25.8	78.5	17.2	

**Table 5:** Summary of the stability enhancement of OSCs by using solid additives.

Additive Type	Active Layer	Stability Mode	Conditions	Key Stability Outcome	PCE	Reference
PQ	PM6:Y6	Thermal stability	85 °C, 160 h	93% PCE retained, reduced energy disorder and suppressed phase segregation	15.2%	[51]
FcF_10_	PM6:Y6	Thermal stability	85 °C, 360 h	88% PCE retained, reduced bimolecular and trap-assisted recombination	17%	[22]
DBP	PM6:L8-BO	Thermal/photostability	1 sun, 1000 h of storage	82% PCE retained, enhanced crystallinity and optimal phase separation morphology	17.7%	[24]
Y6	PM6:PY-DT	Thermal stability	1 sun	T80 lifetime of 1180 h, increased molecular packing, decreased trap density and enlarged phase separation size	18.02%	[52]
DTC-C12	PM6:PY-C11	Photostability/mechanical	1 sun	Maintained 93.6% of its initial efficiency after 3000 consecutive bending cycles, T80 lifetime of 1120 h; suppressed molecular aggregation	18.3%	[53]
3,6TTBr	PM6:L8-BO	----------------------------	------------------	Reduced trap density and improved carrier transport	20.1%	[54]
BA	PM6:Y6	Photostability	1 sun	80% of the initial PCE retained after 200 h, controlled the film morphology and improved crystallinity	18.5%	[55]
DBF	PM6:L8-BO	Thermal/storage stability	1000 h storage	Suppressed burn-in, high Tg effect	18.6%	[56]
BCN	D18:L8-BO	Storage	960 h storage	96.3% PCE retained, optimized phase separation and refined fiber network	18.2%	[57]
DBM	PM6:Y6	Thermal stability Storage stability	85 °C, 200 h 1000 h	Maintained 80% PCE after thermal annealing at 85 °C for 200 h Maintained 95% of initial PCE	18.7%	[46]
N2200 DTT	PTQ10:Y6	Operational stability	150 °C, 20 h	90% PCE retained (non-volatile) 79% PCE retained (volatile)	18%	[58]
DIB	PM6:Y6	Photostability	30 days (N_2_)	92% PCE retained, improved crystallinity, reduced charge recombination and showed superior phase separation	16.6%	[45]
PyMC5	PM6:L8-BO	Thermal and photostability	Tested under 1 sun placed in 65 °C and heat exposure for 500 h	Maintained 83% initial PCE under photostability and 90% initial PCE under thermal stability	19.5%	[28]
PID	PM6:Y6	Thermal stability	85 °C, 1200 h	Retained 98% of initial PCE (after elimination of burn-in loss)	16.3%	[20]

## Data Availability

Data sharing is not applicable. No new data were created or analyzed in this study.
